# Vallecular Cysts in Newborns: A Case Series Demystifying the Obscured Anomaly

**DOI:** 10.7759/cureus.57626

**Published:** 2024-04-04

**Authors:** Noorain Nadhrah Muslim, Nadhirah Mohd Shakri, Santhi Kalimuthu, Shashi Gopalan, Puteri Nur Baiduri Zainal Abidin

**Affiliations:** 1 Otolaryngology - Head and Neck Surgery, Universiti Kebangsaan Malaysia (UKM), Kuala Lumpur, MYS; 2 Otolaryngology - Head and Neck Surgery, Hospital Tengku Ampuan Rahimah, Klang, MYS

**Keywords:** newborn, stridor, laryngoscopy, laryngeal cyst, vallecular cyst

## Abstract

A vallecular cyst is a rare diagnosis in newborns presented with stridor, which poses a significant threat to the well-being of infants. This potentially life-threatening condition is associated with a range of complications, including respiratory distress, feeding difficulties, and failure to thrive. Through this case series, we aim to shed light on the suspicion of vallecular cysts in newborns presenting with stridor and the complexities encountered during their management, highlighting the importance of early recognition and intervention. We presented a case series consisting of three newborns who presented with stridor and respiratory distress symptoms to our center. All three cases were diagnosed using a flexible laryngoscope, and surgical intervention was done. The vallecular cyst was removed, and subsequent follow-up showed no recurrence of the lesion. This case series highlights the importance of early suspicion and recognition of vallecular cysts in newborns, emphasizing the thorough examination during diagnostic evaluations. Proper surgical planning and appropriate ventilation strategies are essential for the successful management and resolution of symptoms.

## Introduction

The incidence rate of vallecular cysts ranges from 1.82 to 3.49 per 100,000 live births [1]. Albeit rare, their potential to cause respiratory distress and compromise the airway necessitates a thorough understanding of their clinical presentation, diagnostic methods, and treatment approaches [2]. Situated at the vallecula, clinical features of vallecular cyst include stridor, respiratory distress, feeding difficulties, and failure to thrive [1-3]. Larger lesions may lead to life-threatening airway obstruction, which poses significant morbidity and mortality if not promptly recognized and managed. In this case series, our objective is to illuminate the suspicion of vallecular cysts in newborns who present with stridor, as well as to explore the intricacies involved in their management. By doing so, we emphasize the criticality of early recognition and intervention in addressing this condition effectively.

## Case presentation

Case 1

**Figure 1:**
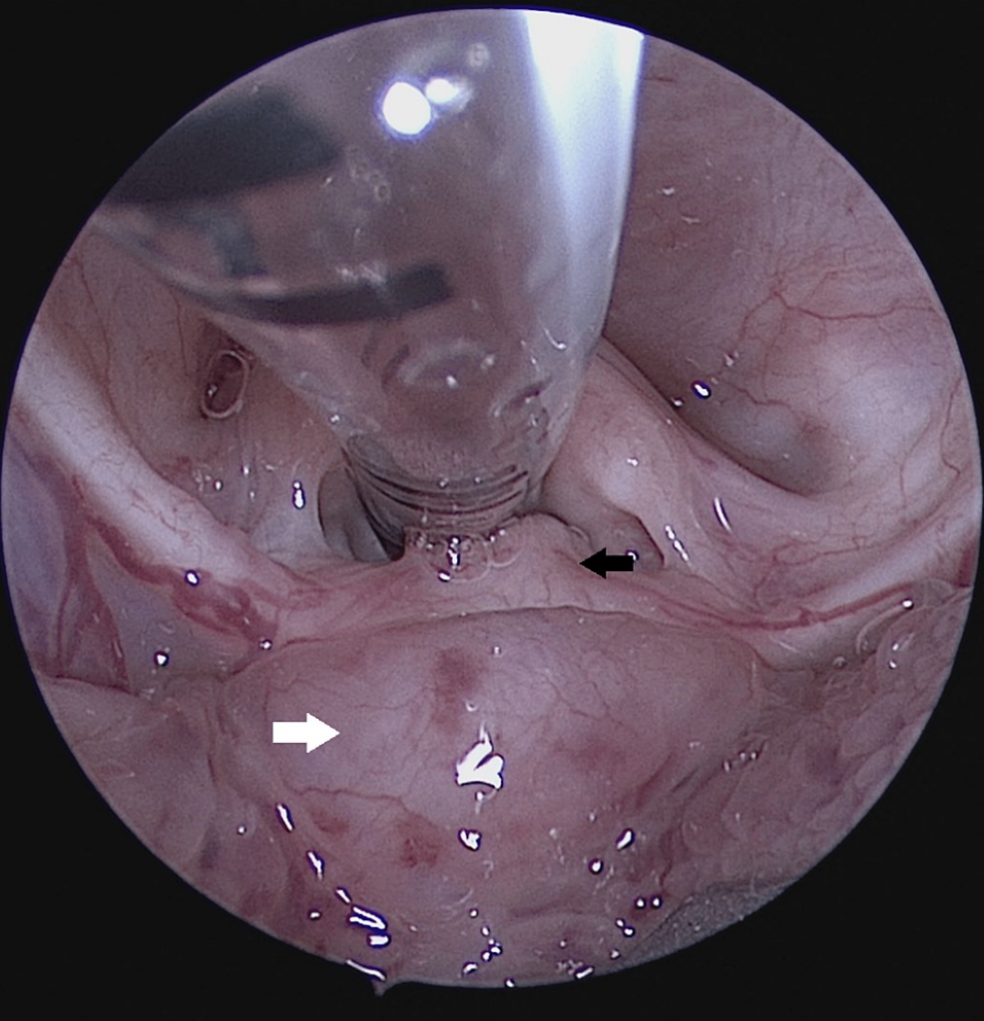
Direct laryngoscopy view of the larynx showing a vallecular cyst (white arrow) pushing the epiglottis (black arrow) posteriorly.

**Figure 2:**
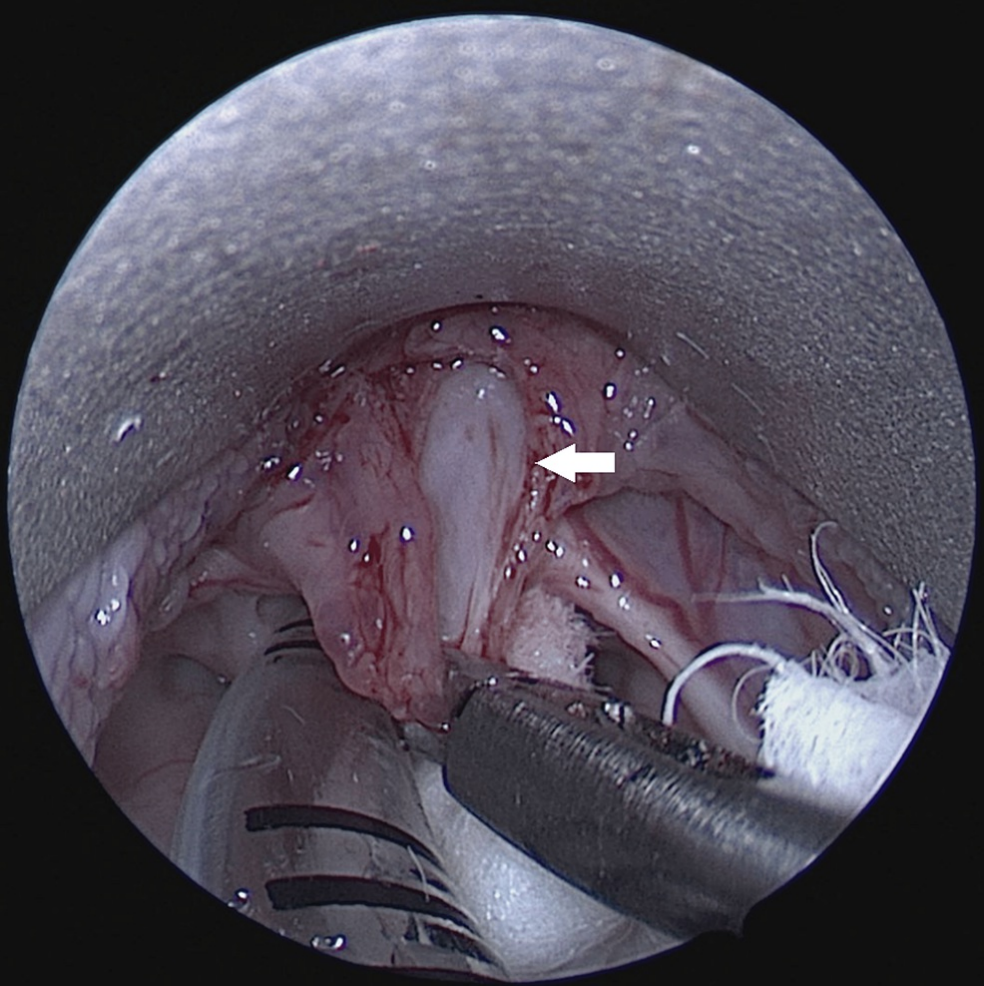
Direct laryngoscopic view of the vallecular cyst excision. Cyst wall (white arrow) identified and excised completely.

A 44-day-old term girl was referred for progressively worsening stridor and chest recession for two weeks. Flexible nasopharyngolaryngoscopy (FNPLS) revealed a cystic lesion at the vallecula, pushing the epiglottis posteriorly, compromising the airway. During direct laryngoscopy, the infant experienced difficulties in maintaining good oxygen saturation on positive-pressure ventilation by mask. The cyst was then punctured and aspirated using a 20 gauge branula connected to a 1cc syringe. Reduction of the cyst size allowed better exposure of the glottis for intubation (Figure 1). She subsequently underwent complete excision of the vallecular cyst using the cold method (Figure 2). Post-operatively, the baby showed resolution of symptoms, and subsequent examinations during clinic follow-up confirmed no sign of recurrence.

Case 2

A term one-month-old boy presented with stridor associated with intermittent cyanotic episodes for three weeks. His feedings were frequently interrupted, leading to poor weight gain. Examination showed inspiratory stridor with the presence of suprasternal recession and subcostal recession. Bedside FNPLS revealed a vallecular cyst pushing the epiglottis posteriorly, with features of laryngomalacia, including redundant arytenoid mucosa and short aryepiglottic folds. The cyst was aspirated prior to intubation, and the patient underwent direct laryngoscopy, tracheoscopy, marsupialization of the vallecular cyst, and supraglottoplasty using the cold method. The surgery was uneventful, and subsequently the patient showed improved feeding tolerance, increased weight gain, and complete resolution of symptoms.

Case 3

**Figure 3:**
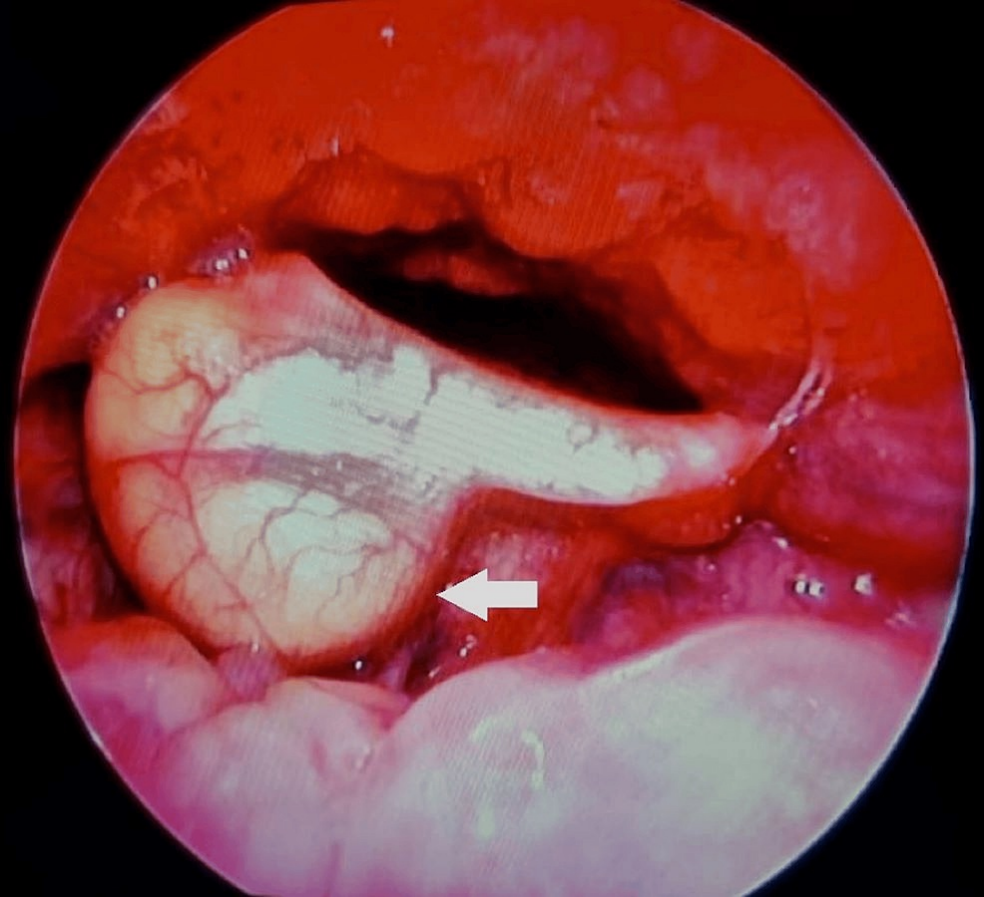
Flexible endoscopy view of the vallecular cyst (white arrow).

A two-month-old infant presented with a gradual onset of stridor and rapid breathing for one month. The patient also experienced interrupted feeding with poor weight gain, but no cyanotic episode was observed. FNPLS revealed a vallecular cyst along with bilateral redundant mucosa of the arytenoid, short aryepiglottic fold, and epiglottis flopping into the laryngeal inlet (Figure 3).

Marsupialization of the vallecular cyst and supraglottoplasty were performed, and intraoperatively the cystic mucous material was suctioned out. Subsequent clinic follow-ups showed no evidence of residual or recurrence of the vallecular cyst.

## Discussion

Vallecular cyst is part of a broader category of benign cystic lesions in the larynx. De Santo's classification of laryngeal cysts includes ductal cysts, saccular cysts, and thyroid-cartilage foraminal cysts. Among these, a ductal cyst is the most commonly observed type and typically presents as a retention cyst due to obstruction of seromucinous glands in the laryngeal mucosa. It can manifest in various regions in the larynx, with vallecula and true cords being the most frequent sites [4]. Infants with vallecular cysts are most commonly diagnosed between birth and 16 weeks of age, with a mean age of 40 days of life at diagnosis [3].

Despite its rare occurrence, a vallecular cyst has profound impacts on the patients due to its potential to cause upper airway obstruction. The clinical presentations include inspiratory stridor, chest recessions, feeding difficulties leading to failure to thrive or cyanosis, and apnea in severe cases [5]. All of our patients presented with symptoms of upper airway obstruction. A progressively enlarging cyst may elevate inspiratory negative pressures, contributing to supraglottic prolapse. Subsequent altered respiratory mechanics and negative intrathoracic pressures caused by partial obstruction lead to gastroesophageal reflux (GER), which results in feeding difficulties [6]. Interrupted feeding will ultimately impair the growth and development [7]. Thus, early recognition and intervention are crucial to provide necessary nutrition and optimize the overall well-being of infants. Two of our patients exhibited feeding difficulties, resulting in poor weight gain consistently below the 3rd percentile on the growth chart.

It is worth noting that vallecular cysts may coexist with other laryngeal anomalies, including laryngomalacia, which is the most common cause of neonatal stridor characterized by floppy supraglottic structures. The progressive enlargement of vallecular cysts alters the airway dynamics, leading to increased inspiratory negative pressures that contribute to supraglottic prolapse and the development of laryngomalacia [8]. Although most infants with laryngomalacia outgrow the symptoms without requiring operative intervention by 24 months of age, clinical presentation can be more complicated when it coexists with vallecular cysts [1]. It results in altered respiratory mechanics, negative intrathoracic pressures, gastroesophageal reflux, feeding difficulties, and further airway compromise [8].

FNPLS serves as a valuable tool in diagnosing vallecular cysts. It allows dynamic visualization of the larynx and identification of the cyst within the vallecula. FNPLS enables a detailed assessment of the cyst's size, location, and anatomical relationship with surrounding structures. By accurately diagnosing vallecular cysts, surgeons can plan the subsequent steps of management more effectively [8]. Imaging modalities, such as computed tomography and magnetic resonance imaging, may be warranted pre-operatively to delineate the cyst’s structure, extension, and vascularity and to identify the presence of other lesions such as hemangioma, thyroglossal duct cysts, lingual thyroid, and lymphatic malformation. However, in our specific case series, FNPLS alone was found to be sufficient to diagnose vallecular cysts, eliminating the need for additional imaging studies.

With the advancement of technology, prenatal diagnosis of vallecular cysts is possible through detailed ultrasound examinations during the second trimester of pregnancy. As described by Cuillier et al., ultrasonography done in the neck area during the second trimester of pregnancy is preferable and more informative because the fetal head is not yet in a flexed position and imaging can usually be carried out from several angles [9]. Identification of vallecula cysts before birth provides valuable information for parents counseling, delivery planning, and organizing a multidisciplinary team for immediate postnatal care. During delivery, the placental cord can be left undivided until the airway is secured, permitting complicated procedures such as operation on placental support, which may involve an elective Cesarean section under tocolysis while maintaining oxygen supply through the placenta [10].

Treatment of vallecular cysts can vary depending on the size, location, and symptoms of the cyst. Conservative management may be appropriate for small and asymptomatic cysts. Larger cysts with significant airway obstruction or persistent symptoms require surgical intervention. Multiple surgical intervention techniques have been reported in treating vallecular cysts, such as marsupialization, endoscopic excision, laser ablation, and coblator-assisted cyst excision [8]. Large cysts are more challenging to treat as they may obscure the laryngeal inlet and cause difficulty in securing the airway prior to surgical intervention. Case 2 was pre-oxygenated with 100% oxygen under spontaneous ventilation and given intravenous sedation before proceeding with direct laryngoscopy. However, the patient desaturated down to 38% during the procedure, preventing the surgeon from proceeding with tracheoscopy. Good oxygenation was established by the laryngeal mask airway. An attempt for endotracheal intubation was done by an anesthesiologist but failed due to poor visualization of the glottis. The cyst was then aspirated using a 20 gauge branula connected to a 1cc syringe in order to reduce the cyst size, allowing successful intubation and subsequently complete excision of the vallecula cyst. We would like to emphasize good communication between surgeons and anesthesiologists is essential, including pre-operative planning and meticulous intraoperative approach to maintain optimal oxygenation and ventilation. The role of aspirating the cyst is controversial as it may increase the risk of cyst rupture. However, it is permissible during emergencies such as airway obstruction to assist in intubation prior to cyst removal.

## Conclusions

Diagnosing vallecular cysts demands a high level of clinical suspicion. Vallecular cysts should be one of the differential diagnoses of newborns with inspiratory stridor. Early recognition and intervention are paramount in preventing serious consequences, reducing the risk of long-term respiratory and feeding difficulties.

With appropriate management, most newborns with vallecular cysts can achieve favorable outcomes. Follow-up evaluations are equally important to monitor symptoms and recurrence and to ensure appropriate growth and development.
